# Effect of black chokeberry on skeletal muscle damage and neuronal cell death

**DOI:** 10.20463/jenb.2019.0028

**Published:** 2019-12-31

**Authors:** Jisu Kim, Kang Pa Lee, Suji Beak, Hye Ra Kang, Yong Kyun Kim, Kiwon Lim

**Affiliations:** 1 Department of Sports Medicine and Science in Graduated School, Konkuk University, Seoul Republic of Korea; 2 Physical Activity and Performance Institute (PAPI), Konkuk University, Seoul Republic of Korea; 3 Department of Bio-Science, College of Natural Science, Dongguk University Republic of Korea; 4 Department of Radiological Science, Daewon University college, Chugbuk Republic of Korea; 5 Department of Physical Education, Konkuk University, Seoul Republic of Korea

**Keywords:** *Aronia melanocapa* L, black chokeberry, Alzheimer's disease, Parkinson’s disease, exercise supplement

## Abstract

**[Purpose]:**

Numerous epidemiological studies have shown that it is possible to prescribe exercise for neurodegenerative disease, such as Alzheimer's disease and Parkinson’s disease. However, despite the availability of diverse scientific knowledge, the effects of exercise in this regard are still unclear. Therefore, this study attempted to investigate a substance, such as black chokeberry (*Aronia melanocapa* L.) that could improve the ability of the treatment and enhance the benefits of exercising in neurodegenerative diseases.

**[Methods]:**

The cell viability was tested with 2,3-bis[2-methyloxy-4-nitro-5-sulfophenyl]-2H-tetrazolim-5-carboxanilide and the cells were stained with ethidium homodimer-1 solution. The mRNA expression levels were evaluated by microarray. The active compounds of black chokeberry ethanolic extract (BCE) were analyzed by gas chromatography. The chemical shift analysis in the brain was performed using magnetic resonance spectroscopy.

**[Results]:**

BCE treatment decreased hydrogen peroxide-induced L6 cell death and beta amyloid induced primary neuronal cell death. Furthermore, BCE treatment significantly reduced the mRNA levels of the inflammatory factors, such as IL-1α, Cxcl13, IL36rn, Itgb2, Epha2, Slamf8, Itgb6, Kdm6b, Acvr1, Cd6, Adora3, Cd27, Gata3, Tnfrsf25, Cd40lg, Clec10a, and Slc11a1, in the primary neuronal cells. Next, we identified 16 active compounds from BCE, including D-mannitol. *In vivo*, BCE (administered orally at a dosage of 50 mg/kg) significantly regulated chemical shift in the brain.

**[Conclusion]:**

Our findings suggest that BCE can serve as a candidate for neurodegenerative disease therapy owing to its cyto-protective and anti-inflammatory effects. Therefore, BCE treatment is expected to prevent damage to the muscles and neurons of the athletes who continue high intensity exercise. In future studies, it would be necessary to elucidate the effects of combined BCE intake and exercise.

## INTRODUCTION

Neurodegenerative disorders, such as Alzheimer's disease (AD) and Parkinson’s disease, have physiological characteristics that are associated with memory loss or stiffness of movement^1^. The mechanisms underlying the pathogenesis of neurodegenerative disorders are interrelated in complex vicious circles, such as oxidative stress, mitochondrial dysfunction, DNA damage, fragmentation of neuronal Golgi apparatus, and neuro-inflammation^2^. Typically, the anatomical characteristics of neurodegeneration include accumulation of the beta-amyloid (Aβ) peptide and neuronal cell death^3^. Therefore, the amyloid cascade hypothesis suggests that inflammatory responses consequently induce neurodegenerative diseases to increase free radical species and induce neuronal damage^4^.

Reactive oxygen species (ROS) are involved in various etiological factors^5^. For example, the progression of neurodegenerative diseases is accelerated by inflammatory reactions and generation of reactive oxygen species^6,7^. Additionally, excessive oxidative stress and inflammation are engaged in causing irreversible cell damage in the muscle fiber^8^. Therefore, exercising at the therapy stage is required for blocking ROS generation and modulating the inflammatory mediators. However, considering the beneficial effects of ROS in vivo, ROS produced by exercising is known to help neurogenesis by upregulating the expression of brain-derived neurotrophic factor (BDNF)^9^. It is possible to develop exercise as an alternative therapy for neurodegeneration. For example, regular exercise is beneficial for the activation of brain functioning and may have a protective effect on stroke and AD. Thus, the research trends in the sports science are inclined not only towards enhancing the exercise performance without muscle damage but also to modulate neuroplasticity for the patients with neurodegeneration^10,11^.

Black chokeberry has excellent free radical scavenging activity, which plays an important role in anti-aging^12,13^. In particular, the plant has polyphenolic compounds, such as flavanone, anthocyanin, and isoflavone, which provide strong antioxidant and physiological activities based on radical scavenging abilities^14,15^. Previously, Lee et al, have reported that black chokeberry extracts can exert neuroprotection by regulating the inflammatory response factors in the amyloid beta accumulation pathway in the brain^16^. However, the report was lacking in the predictive aspect of the effectiveness of prescribing black chokeberry in combination with exercise in the patient with neuronal disease.

Therefore, this study investigated the compounds present in black chokeberry that can exert its pharmacological and biological activities that are thought to inhibit AD development or progression using gas chromatography. Thus, we investigated the response of various genes to black chokeberry fruit extracts in Aβ-stimulated neuronal cells through microarray analysis. In addition, we confirmed the cyto-protective effects of the black chokeberry fruit extracts in Aβ-induced primary neuronal cell death and hydrogen peroxide-induced skeletal muscle cell death.

Our findings suggest that black chokeberry fruit extracts can serve as a functional food containing potential neuroprotective components, which can be transmitted across brain barriers. We also suggest that this extract contains a potential agent that can be used as sports supplements, and it can not only help treat patients with neurodegenerative diseases but can also contribute to the prevention of muscle damage.

## METHODS

### Kits and reagents

Materials for cell culture and Live/Dead TM Viability/Cytotoxicity kit were purchased from Thermo Fisher Scientific (Waltham, MA, USA). The 2,3-bis-(2-methoxy-4-nitro-5-sulfophenyl)-2h-tetrazolium-5-carboxanilide (XTT) kit was obtained from Welgene Inc. (Gyeonsan-si, Gyeonsanbuk-do, Republic of Korea). All other reagents were purchased from Sigma-Aldrich (St. Louis, MO, USA).

### Preparation of ethanolic extract from black chokeberry fruit (BCE)

Black chokeberry extracts were obtained as described previously, with slight modifications^15^. Black chokeberry fruit (200 g) was blended, and then the fine powder was extracted using 1,000 mL of ethanol for 24 h. The ethanolic extracts were concentrated by evaporation at 60°C in vacuum. The precipitate was dissolved in sterile deionized water (50 mL). The aqueous extract was lyophilized by freeze-drying at -60°C.

### Cell culture

Cells were cultured as previously described^14^. Rat skeletal muscle cells (L6 cells) were purchased from the Korean Cell Line Bank (Seoul, Korea) and grown in Dulbecco’s modified Eagle’s medium (DMEM) containing 10% fetal bovine serum and 1% penicillin-streptomycin at 37±2°C and 5% CO_2_. The primary neuronal cells were isolated from prenatal Sprague-Dawley rats and were grown in a neurobasal medium containing 1% L-glutamine, 1% PS, and B27 supplement for 7 days at 37±2°C and 5% CO_2_.

### Cell viability

To determine cell viability, the cells were treated with XTT reagent or ethidium homodimer-1 (EthD-1) solution. First, the cells were seeded in a 96-well plate and then treated with hydrogen peroxide or amyloid beta (Aβ 25-35) or BCE. The cells were incubated with XTT dye for 2 h, and the formazan dye was measured using the microplate reader at 450 nm. Next, the cells were incubated with 10 μM of EthD-1 solution for 20 min at 25±2°C. The images were captured using the fluorescence microscope.

### Target labelling and hybridization of microarray

To analyze the gene expression profiles related to the protective effect of BCE in neurodegeneration, microarray analysis was performed as previously described, with slight modifications^17^. Briefly, the hybridization images were analyzed by a DNA microarray scanner (Agilent Technology, USA), and the data were quantified using Agilent Feature Extraction software 10.7 (Agilent Technology, USA). The average fluorescence intensity for each spot was calculated and the local background was subtracted. Data normalization and selection of fold-change genes were performed using GeneSpringGX 7.3.1 (Agilent Technology). Genes were filtered by removing flag-out genes in each experiment. Intensity-dependent normalization (LOWESS) was performed, where the ratio was reduced to the residual of the Lowess fit of the intensity vs. ratio curve. The average of the normalized ratio means was calculated by dividing the average of the normalized signal channel intensity by that of the normalized control channel intensity. The functional annotation of genes was performed according to Gene OntologyTM Consortium (http://www.geneontology.org/index.shtml) using GeneSpringGX 7.3.

### Gas chromatography-mass spectrometry (GC/MS) analysis

GC/MS analysis was performed as previously described^18^, using an Agilent 6890N GC/5975i MS instrument (Palo Alto, CA, USA) and DB5-MS capillary column (30 m × 250 μm, 0.25 μm film thickness). Helium was used as the carrier gas at a flow rate of 1 mL/min. The injector port and interface temperatures were 280°C and 300°C, respectively. The gas chromatography oven was maintained at 40°C for 2 min and increased to 230°C at a rate of 5°C/min, and then kept constant at 300°C for 5 min. The split ratio was 1:10; the mass ranges were from 40-800 m/z.

### Animal care and in vivo studies

The animal care and in vivo studies were performed as previously reported^19,20^. Male ICR (Hsd:ICR (CD-1®) ;7 weeks old; 22 ± 2g; n= 4) were purchased from Orient Bio (Seongnam, Korea). This study was conducted in accordance with the ethical guidelines of the Konkuk University Institutional Animal Care and Use Committee, which incorporates the guidelines put forth in the Declaration of Helsinki (1964). The mice were individually housed with ad libitum access to water and food (AIN 93G formula). All mice were kept in a controlled environment (room temperature, 24°C ± 2°C; humidity, 40% ± 2%; 12 h light/ dark cycle). To perform the MRI analysis, all mice were tested sequentially, and the MRI images of the brain were obtained after oral administration of saline to the mice. Immediately after image acquisition, BCE was orally administered to the same mice and the MRI images were obtained.

### Magnetic resonance spectroscopy

To acquire the MRI images, all mice were anesthetized with 1.5% isoflurane in a 3:7 mixture of oxygen and nitrogen dioxide. The respiration rate and body temperature of the experimental animals were maintained using an animal monitoring-gating system (SA instruments, Stony Brook, NY, USA) and warm bed, respectively. MRI imaging experiments were performed on a Bruker 4.7 T (Bruker, Ettlingen, Germany) horizontal bore magnet using a transmitter coil with an inner diameter of 86 mm and a 4-channel mouse brain surface coil. The magnetic resonance spectroscopy (MRS) was used to measure the repetition time (TR = 2500 ms), echo time (TE = 17 ms), number of excitations (NEX = 256), and the acquisition point for a single voxel of 1.7 mm × 2 mm × 2.2 mm, using a PRESS sequence of 2048. Analysis of metabolite changes using 1H-MRS was performed using the LCmodel software (Stephen Provencher, Canada).

### Statistical analysis

Statistical analysis was performed as previously described^21^. The results are expressed as the mean ± standard error of at least three independent experiments (n ≥ 3). Between-group differences were determined using Student’s t-test and one-way analysis of variance. Tukey’s test was used for multiple comparisons (GraphPad Prism ver. 4.00 for Windows, La Jolla, CA, USA). P < 0.05 was considered statistically significant.

## RESULTS

### Effects of BCE on exogenous hydrogen peroxide-stimulated L6 skeletal muscle cells

**Figure 1.**
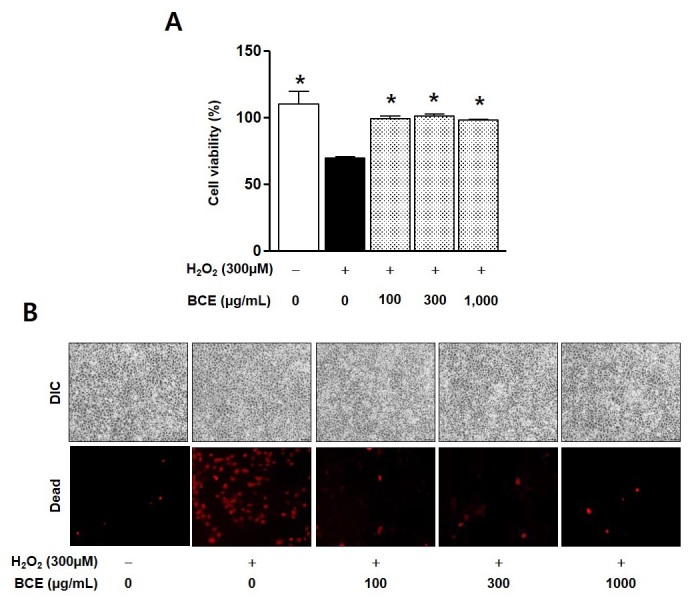
Cytoprotective effects of BCE treatment on H_2_O_2_-stimulated L6 cells. L6 cells (1 × 10^5^) were treated with hydrogen peroxide (H_2_O_2_, 300 μM), with or without Black chokeberry ethanol extract (BCE: 100, 300, and 1000 μg/mL) for 24 h. (A) The cells were incubated with 2,3-bis-(2-methoxy-4-nitro-5-sulfophenyl)-2H-tetrazolium-5-carboxanilideat 37°C for 4 h. Absorbance was measured at 450 nm using a plate reader. Bar graphs indicate the percentage of cell viability. Data are expressed as the mean ± SE values from three independent experiments. *P < 0.05 compared to only H_2_O_2_ treated group. (B) The Live/Dead cell reagents were dispensed into each well, and these images were acquired using a fluorescence microscope (excitation 580 nm and emission 604 nm).

To determine whether BCE could affect the cellular responses to exogenous H_2_O_2_ in L6 cells, the cytotoxic effects of the BCE treatment (100-1,000 μg/mL) on L6 cells, in the presence or absence of H_2_O_2_, were tested using the XTT assay. The BCE treatment did not affect L6 cell viability up to 1,000 μg/mL (n = 3; Fig. 1A). H_2_O_2_ (300 μM) treatment reduced L6 cell viability, and this response was inhibited in the BCE-treated L6 cells (n = 3; Fig. 1B).

### Effects of BCE on hippocampus in ICR mouse Brain

**Figure 2.**
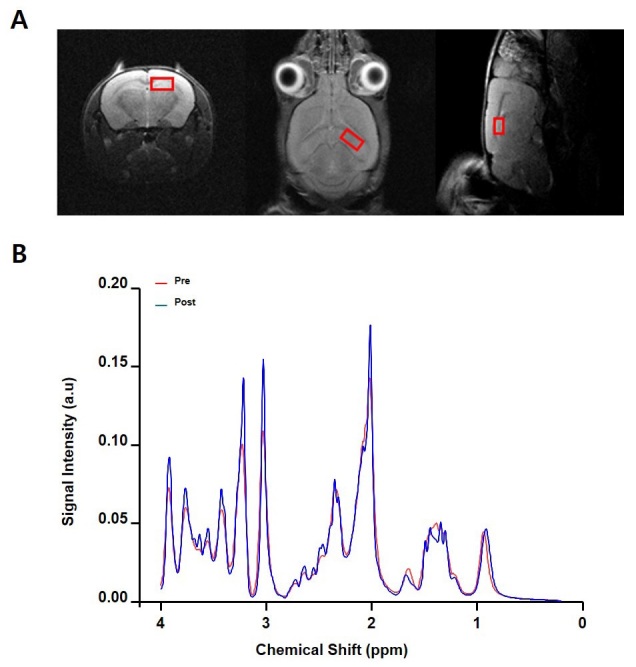
Analysis of MRI and MRS in mouse brains after oral administration of BCE. (A) Representative images from the hippocampal MRI performed on a black chokeberry ethanol extract (BCE)-treated mouse (weight: 50 mg/kg). (B) In the spectra originating from the region, the shift in the chemical profiles before and after administration of BCE is displayed.

**Figure 3.**
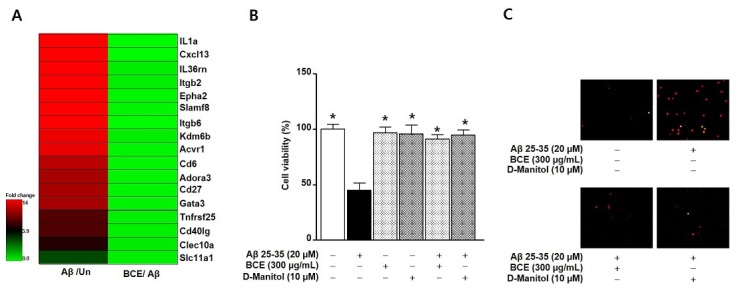
Transcriptomic profiles and cell viability in response to BCE treatment in Aβ-induced primary neuron cell death. (A) Hierarchical clustering of microarray data obtained from BCE-treated (with 300 μg/mL) amyloid beta (Aβ)-stimulated primary neuronal cells. (B) The primary neuronal cells (1 × 10^5^) were treated with Aβ 25-35 (20 uM), with or without BCE (300 μg/mL) for 24 h. Bar graphs indicate the percentage of cell viability. Data are expressed as the mean ± SE values from three independent experiments. *P < 0.05 compared to only Aβ 25-35 treated group. (C) The Live/Dead cell reagents were dispensed into each well, and these images were acquired using a fluorescence microscope (excitation 580 nm and emission 604 nm). Un: untreated group; BCE: black chokeberry ethanol extract treated group; Aβ: amyloid β treated group

To test whether the BCE treatment had an effect on the hippocampus in the mouse brain, we analyzed the brain function using MRI and MRS. Figure 2A shows representative images of the MRI of the hippocampus in a BCE-treated mouse. The first set of MRI images was acquired of all the mouse brains, and the second set of MRI images was acquired in the same mice after oral administration of BCE (50 mg/kg). As shown in Figure 3B, MRS was used to measure the TR = 2500 ms, TE = 17 ms, NEX = 256, and acquisition point for a single voxel of 1.7 mm × 2 mm × 2.2 mm using a PRESS sequence = 2048. The chemical shift in the hippocampus in ICR mouse brain indicates a difference between the untreated and BCE-treated mouse brain.

### Analysis of BCE composition

The BCE composition was determined using GC/ MS analysis. As depicted in Table 1, BCE contains the following 16 compounds: formic acid (1.82%), butyl ketone (0.61%), 2-pyrone-4-D 5-hexen-2-one (2.1%), diaziridine (0.93%), benzoic acid (0.5%), 1H-pyrazole, 4,5-dihydro-3-methyl-1-propyl- (0.61%), cyclopentanol (9.13%), chloromethyl 4-chlorododecanoate cyclopentadecanone, 2-hydroxy- (0.45%), sorbitol (0.89%), 2-deoxy-D-ribose (0.43%), 2,4-pentadienoic acid (0.81%), 2-thiazolidinimine (0.53%), 2-deoxy-D-galactose (0.79%), quinic acid (10.77%), and D-mannitol (25.25%).

**Table 1.** GC-MS peak report of ethanol extract of black chokeberry.

No.	RT	Compound name	Peak Area(%)
1	3.169	DIMETHYL PHOSPHINE; (CH3)2PH Formic acid, 2-methylpropyl ester	1.82
2	4.402	Butyl ketone	0.61
3	5.668	2-PYRONE-4-D 5-Hexen-2-one	2.1
4	6.491	Diaziridine,3,3-dimethyl-; 3,3-Dimethyldiaziridine Propanal	0.93
5	6.819	Benzoic acid	0.5
6	9.779	1H-Pyrazole, 4,5-dihydro-3-methyl-1- propyl-	0.69
7	10.831	Cyclopentanol	9.13
8	11.095	Chloromethyl 4-chlorododecanoate Cyclopentadecanone, 2-hydroxy-	0.45
9	11.374	Sorbitol	0.89
10	11.506	2-Deoxy-D-ribose	0.43
11	11.637	2,4-PENTADIENEOIC ACID 1,3- Butadiene-1-carboxylic acid	0.81
12	12.048	2-Thiazolidinimine, 3-methyl-3- ethyl-1-thia-cyclopentane 3-Ethylthiolane	0.53
13	12.377	2-Deoxy-D-galactose	0.79
14	13.051	Quinic acid	10.77
15	15.271	D-Mannitol	25.25
16	15.699	Glucitol	39.45

### Neuroprotective effects of BCE and D-mannitol are mediated through downregulation of inflammatory factors

We investigated the protective effects of BCE on Aβ-induced primary neuron cell death by XTT assay and microarray profiling. First, the mRNA was isolated from the neuronal cells after treatment with BCE (300 μg/mL) or Aβ (20 μM) for 24 h. Next, to identify the genes altered by treatment of neurons with Aβ, a gene microarray analysis was performed. Figure 3A indicates the expression of the differentially expressed genes (>1.3 fold). A total of 6,433 genes were found to be altered between the untreated and Aβ-stimulated groups. There was an overlap of 17 genes from the two gene categories: cell death and inflammatory response in Aβ-treated primary neuron cells, in the presence or absence of BCE. Aβ treatment induced the expression of IL-1α (Gene ID: 10532), Cxcl13 (Gene ID: 5279), IL36rn (Gene ID: 9571), Itgb2 (Gene ID: 9354), Epha2 (Gene ID: 12950), Slamf8 (Gene ID: 5038), Itgb6 (Gene ID: 9887), Kdm6b (Gene ID: 2899), Acvr1 (Gene ID: 9874), Cd6 (Gene ID: 1957), Adora3 (Gene ID: 8759), Cd27 (Gene ID: 11878), Gata3 (Gene ID: 7070), Tnfrsf25 (Gene ID: 13035), Cd40lg (Gene ID: 17093), Clec10a (Gene ID: 2943), and Slc11a1 (Gene ID: 16229), by more than 2-fold, whereas BCE treatment significantly downregulated the expression of those Aβ-induced genes to 0.19-fold. Next, the XTT assay was used to determine the inhibitory effects of BCE treatment on Aβ-induced primary neuronal cell death. As shown in Figure 3 B and C, the treatment of neuronal cells with BCE (300 μg/mL) or D-mannitol (10 μM) did not significantly alter the cell viability as compared to the untreated group. Aβ (20 μM) significantly decreased the primary neuronal cell viability, whereas BCE (300 μg/mL) and D-mannitol (10 μM) significantly inhibited the Aβ-induced neuronal cell death.

## DISCUSSION

In the present study, we found that BCE treatment can exert a cytoprotective effect in H_2_O_2_-induced skeletal muscle cell death and Aβ-induced neuronal cell death. Moreover, treatment with BCE modulated the chemical shift in the hippocampus of the brain. These results imply that the antioxidant effects of BCE (300 μg/mL) can significantly reduce the H_2_O_2_-induced skeletal muscle cell death. In addition, BCE treatment inhibits Aβ-induced neuronal cell death through anti-inflammatory signals and activation of transcriptional pathways, such as IL-1a, Cxcl13, IL 36rn, Itgb2, Epha2, Slamf8, Itgb6, Kdm6b, Acvr1, Cd6, Adora3, Cd27, Gata3, Tnfrsf25, Cd401g, Clec10a, and Slc11a1. Furthermore, the oral administration of BCE (50 mg/kg) exhibits the regulating brain chemical composition. Therefore, the BCE may be a potential supplemental agent for the patients.

In the current study, our hypothesis was that the various bioactive substances in black chokeberry may improve the benefits of exercise on neurodegeneration. It is well known that mannitol contributes greatly to the influx of BNDF into the brain^22^. Combining anaerobic and aerobic exercise for the health of the body can contribute to the activation of neuroplasticity. Exercising has been reported to be effective in activating BNDF^23,24^. Several previous studies have demonstrated the differential expression of BNDF between the athletes and non-athletes. Furthermore, BDNF expression levels have been found to be higher in the healthy group than in the stroke patient group, who are unable to exercise^25,26^. In this study, we observed the presence of large amounts of mannitol in BCE (Table 1). Furthermore, our results indicated that BCE can regulate the chemical composition of hippocampus in the mouse brain (Figure 2). Therefore, we speculated that BCE may contain compounds that can change the metabolites in the brain hippocampus region. Therefore, our data suggest that BCE treatment during exercise may have synergic effects to cause the increase in BDNF expression levels.

Ang et al reviewed the beneficial effects of exercising on neurogenesis and neurodegeneration^27^. Although regular exercise and nutritional intake have been shown to improve health, its application for patients with neurodegenerative diseases still has still not been proven. If patients with neurodegenerative disorders attempt excessive exercise, they may risk injuries. Therefore, when prescribing exercise to patients with neurodegenerative diseases, exposure to the risk of injury should be considered, as these groups may need supplements for the treatment of brain diseases and defense against muscle damage^27^. It is well known that quinic acid is a strong scavenger in vitro and *in vivo*^28^. In present study, we also found that BCE contained compounds, such as quinic acid and D-mannitol (Table 1). Further, BCE exhibited anti-inflammatory activities against Aβ-stimulated neurons (Figure 3) and antioxidant activities against H_2_O_2_-induced skeletal muscle cell death (Figure 1). Therefore, we suggest that BCE can potentially be useful for improving neurodegeneration through its protective effect against oxidative stress-induced cell death.

In conclusion, we suggest that BCE is a potential candidate for treatment of neurodegeneration because of its ability to reduce Aβ-induced neuronal cell death through the modulation of the inflammation-related signaling pathways. Furthermore, our study focused on the ability of free radical scavenging activity in skeletal muscle damage. Therefore, BCE is a promising candidate as a therapeutic agent against Aβ-mediated neurodegeneration, and against muscle damage by excessive exercise. In future studies, it would be necessary to elucidate the effects of combined BCE intake and exercise.
